# Fucose metabolism as a central axis linking inflammation, immunity, and cancer in the gut

**DOI:** 10.3389/fimmu.2026.1898566

**Published:** 2026-07-22

**Authors:** Dhirendra K. Singh, Chieko Saito, Yukihiro Yamaguchi, Olivia G. Cassidy, Keita Nishiyama, Lei Huang

**Affiliations:** 1 Department of Rheumatology and Inflammation Research, University of Gothenburg, Gothenburg, Sweden; 2 Department of Biology, American University, Washington, DC, United States; 3 Department of Pediatrics, University of North Carolina at Chapel Hill, Chapel Hill, NC, United States; 4 Warren Alpert Medical School of Brown University, Providence, RI, United States; 5 Laboratory of Animal Food Function, School of Agricultural Science, Tohoku University, Sendai, Japan; 6 Faculty of Medical Sciences, Newcastle University, Newcastle upon Tyne, United Kingdom

**Keywords:** colorectal cancer (CRC), fucosidase, fucosylation, fucosyltransferase (FUT), inflammatory bowel disease (IBD)

## Abstract

Fucose metabolism and fucosylation are emerging as central regulators of intestinal epithelial integrity, immune homeostasis, host-microbiota interactions, and tumor progression. Increasing evidence implicates dysregulated fucosylation in the pathogenesis of inflammatory bowel disease (IBD), colorectal cancer (CRC), and necrotizing enterocolitis (NEC). Under physiological conditions, fucosylated glycans maintain mucus barrier stability, microbial symbiosis, and mucosal immune tolerance, whereas disrupted fucosylation promotes barrier dysfunction, dysbiosis, chronic inflammation, and intestinal carcinogenesis. In IBD, altered fucosylation impairs epithelial repair and immune regulation. In CRC, abnormal fucosylation drives oncogenic signaling, metabolic reprogramming, immune evasion, and metastasis while also providing clinically relevant biomarkers and therapeutic targets. In NEC, epithelial fucosylation and human milk oligosaccharides such as 2′-fucosyllactose protect against intestinal injury by enhancing epithelial regeneration, modulating inflammatory signaling, and regulating gut microbial composition. Collectively, these findings identify fucose metabolism linking epithelial biology, immunity, microbiota dynamics, and cancer progression in intestinal disease, highlighting fucosylation pathways as promising targets for diagnosis and therapy.

## Introduction

Inflammatory disorders of the gut, such as inflammatory bowel disease (IBD), colorectal cancer (CRC), and necrotizing enterocolitis (NEC), affect millions of people worldwide and pose a major global health burden. IBD alone affects an estimated 6.8 million people worldwide (1), and CRC accounts for ~1.9 million new cases annually (2). NEC continues to be a serious inflammatory condition of premature infants and causes significant morbidity and mortality. Although these diseases differ in age of onset, tissue context, and clinical outcomes, they share overlapping pathogenic mechanisms, such as host genetic susceptibility, immune dysregulation, and alterations in gut microbiota.

Emerging evidence points to a central yet previously underappreciated role for intestinal epithelial glycosylation as a regulatory layer that links these pathogenic factors. Fucosylation is a dynamic glycan modification playing a central role in maintaining intestinal epithelial barrier integrity, shaping host-microbiota interactions, and regulating mucosal immune responses in gut homeostasis and disease. Fucose-containing glycans on mucins are important for the stability of the mucus layer and for the support of symbiotic microbial colonization in the healthy intestine, providing sites of adhesion and sources of nutrients, and thus maintaining spatial segregation of the microbiota from the epithelium (3, 4). Epithelial fucosylation is carefully controlled by host glycosyltransferases and adapts to microbial and environmental signals, facilitating two-way communication between the host and microbes that strengthens mucosal tolerance.

In inflammatory diseases such as IBD, CRC, and NEC, dysregulation of fucosylation pathways, due to changes in glycosyltransferase expression, inflammatory signaling, and dysbiosis, leads to aberrant glycan remodeling, including decreased or structurally altered fucosylated glycans (3, 4). Such changes disturb the organization of mucins and weaken the mucus barrier, which allows bacteria to contact the epithelial surface and promotes chronic inflammation. Disrupted fucose-dependent glycan-lectin interactions further drive aberrant mucosal immune responses by modulating immune cell activation thresholds (5).

Altered fucosylation of immunoglobulins and serum glycoproteins may systemically modulate immune effector functions and enhance inflammatory signaling through Fc receptor pathways, as pro-inflammatory effects of altered IgG glycoforms have been shown in other inflammatory settings (6). Aberrant fucosylation acts as a mechanistic hub linking epithelial barrier breakdown, microbial dysbiosis, and immune activation that collectively contribute to the development and persistence of intestinal inflammatory diseases.

Although the importance of glycosylation in gut pathology is becoming better appreciated, the precise roles of fucose metabolism, fucosyltransferases, and fucose-containing glycans in intestinal inflammation are still poorly defined. Fucosylation pathways might represent a key mechanistic nexus in IBD, CRC, and NEC due to their dual roles in the maintenance of epithelial barrier integrity and in the regulation of immune-microbial interactions.

Here we review current knowledge about fucose and fucosylation in intestinal inflammation with a focus on epithelial barrier biology, immune regulation and host-microbiota interactions. We further discuss the role of dysregulated fucosylation in disease pathogenesis and highlight its potential as a diagnostic marker and therapeutic target in inflammatory diseases of the gut.

## 1 Fucosylation in IBD

### 1.1 Fucose as a metabolic and immunomodulatory regulator in IBD

Fucosylation plays an essential role in maintaining intestinal homeostasis and is a key player in IBD development through host glycan structures, immune system regulation, microbial metabolism, and host-microbe interactions. The dysregulation of fucose-containing glycoconjugates appears to occur in almost all studies and correlates with the occurrence of inflammatory processes in the intestine, and fucose supplementation tends to have protective or regulatory effects on IBDs.

On the level of intestinal barrier function, previous work has shown that the glycosylation of mucins is significantly affected in IBDs. Both UC and CD patients exhibit reduced levels of fucose in their mucin, which is obtained from whole-gut lavage, suggesting a decrease in the mucin glycosylation state that is likely to reduce its protective properties (7). This idea is strengthened through more recent studies into structural analyses of human MUC2, showing that fecal mucus is rich in fucosylated O-glycans, whose modifications due to IBD have a bearing on the metabolism of bacteria and production of short-chain fatty acids (8). These discoveries support the idea that aberrant mucin fucosylation leads to impaired barrier function and disturbed host-microbe relationships in IBD.

Fucose metabolism is also very much involved in the regulation of immune responses and epithelial healing. In experimental colitis models, supplementation of L-fucose has been shown to have an anti-inflammatory action, involving several pathways. Fucose administration in Muc2-deficient mice suppressed pro-inflammatory cytokine secretion and macrophage influx and induced the response of regulatory T cells through Foxp3^+^ Treg generation. Thus, the involvement of adaptive immunity has been recognized as the major component underlying the anti-inflammatory action of fucose (9). Likewise, fucose promotes the regenerative capacity of the intestinal stem cells by stimulating the AHR/IL-22 signaling pathway (10). Such results demonstrate that fucose does not merely regulate immune responses but promotes regeneration as well. Apart from regulating host immune responses, fucose strongly influences the gut microbiome and its metabolic activities. In DSS-induced colitis, fucose administration corrects dysbiosis and restores bile acid metabolism by restoring the microbiome-bile acid communication loop, an action that cannot be achieved when antibiotics are present, indicating its strict dependence on microbiota (11). Furthermore, fucose may alter microbial activity through the regulation of bacterial metabolism. For instance, it suppresses virulence in *Fusobacterium nucleatum* through the inhibition of homocysteine thiolactone, a pro-inflammatory compound (12). Overall, these findings imply that fucose is a metabolite that acts via regulating the microbiome.

In addition, systemic differences in fucosylation are evident in immune proteins within the bloodstream. Patients with IBD have lower fucosylation of their plasma N-glycomics, along with unique glycan branching and sialylation, which correlates with disease severity and treatment needs (13).

Lastly, there is recent evidence to support the idea that fucose not only affects intestinal microbe metabolism but also has an impact on metabolic pathways throughout the body. In a model of DSS colitis, administration of fucose was associated with increased populations of tryptophan-producing microbes and increased tryptophan concentrations, resulting in improvements in behavioral deficiencies despite no improvement in intestinal pathology (14).

### 1.2 Divergent roles of fucosyltransferases in IBD

Fucosyltransferases (FUTs) and their associated glycosylation products represent a central regulatory layer in intestinal homeostasis (See **Box 1**), linking epithelial biology, immune activation, and host-microbiota interactions in IBD. Among these enzymes, FUT2, FUT8, and related fucosylation pathways have emerged as particularly important determinants of mucosal integrity and inflammatory susceptibility, with context-dependent roles in epithelial protection, immune activation, and microbial ecology.

A consistent feature of IBD is the disruption of epithelial and systemic fucosylation. Early biochemical analyses demonstrated that mucin-associated fucose is reduced in both UC and CD, reflecting altered mucus barrier composition and glycan remodeling (7). More recent large-scale glycomic profiling has further shown that IBD is associated with global reductions in plasma protein fucosylation, alongside disease-specific glycan signatures that correlate with clinical severity and treatment needs (13). At the level of immunoglobulins, IgG Fc glycosylation exhibits complex alterations in IBD, where changes in core fucosylation combined with galactose deficiency define a pro-inflammatory antibody signature associated with disease severity and clinical course (15, 16). In addition, fucosylated β-haptoglobin (β-Hp) is elevated in UC and correlates with mucosal healing and endoscopic remission, supporting its utility as a noninvasive biomarker of intestinal inflammation (17). These findings collectively indicate that dysregulated fucosylation is a systemic hallmark of IBD.

#### 1.2.1 α1,2-fucosyltransferase (FUT2)-mediated barrier protection

Among epithelial fucosyltransferases, FUT2 has been implicated in the development of host-microbe interaction and epithelial barrier properties. FUT2 deficiency leads to alterations in glycosylation patterns and gut microbial ecology by enhancing fucose content at the expense of other types of sugar in epithelia, resulting in an expansion of *Bacteroides thetaiotaomicron* population in NHE3-deficient models (18). On the other hand, epithelial-specific deletion of FUT2 exacerbates colitis and promotes the production of lysophosphatidylcholine, a mediator that promotes intestinal inflammation by changing the gut microbiota (19). Mechanistically, FUT2 deficiency not only disturbs microbial metabolic equilibrium but also activates repair programs by generating inflammatory lipid mediators. Notably, FUT2 is essential for proper epithelial stem cell functions, including maintenance and stress resistance in intestine stem cells. FUT2 regulates the fucosylation of HYOU1, the ER chaperone mediating unfolded protein response signaling to prevent inflammatory injury (20). FUT2 is also important for balancing host immunity. Deletion of epithelial α1,2-fucosylation impacts host-microbial glycan communication and causes alterations in fucosylation of bacteria, affecting IgA-dependent immune surveillance and causing colitis (21). Furthermore, changes in fucosylation patterns caused by FUT2 are critical for regulating immune cell differentiation and the development of intestinal mucosal IgA responses.

Functionally, changing FUT2 activity can help protect against colitis. Signals from diet or gut bacteria can increase FUT2 expression and epithelial fucosylation. This improves the barrier’s strength and mucus function. For instance, malic acid from *Pithecellobium clypearia* boosts FUT2 expression and fucosylation, leading to better barrier function and lower colitis severity (22). Similarly, probiotics like *Akkermansia muciniphila* create metabolites (e.g., N-acetylspermidine) that improve α1,2-fucosylation and support tight junctions, which help reduce inflammation (23). These studies show that FUT2-related fucosylation is influenced by gut bacteria and diet and can be targeted for treatment.

#### 1.2.2 α1,6-fucosyltransferase (FUT8)-driven immune activation

In contrast to the generally protective role of epithelial FUT2, FUT8 plays an important role in immune activation and inflammation. Core fucosylation of T-cell receptors is necessary for effective T-cell receptor signaling, cytokine production, and colitis induction. FUT8 deficiency in T cells significantly reduces experimental colitis (24). Similarly, FUT8-mediated core fucosylation is crucial for CD14-dependent Toll-like receptor 2 (TLR2) and Toll-like receptor 4 (TLR4) signaling in macrophages. Its loss decreases inflammatory cytokine production and protects against DSS-induced colitis (25). These findings show that FUT8 boosts both adaptive and innate immune activation, which can lead to increased intestinal inflammation when overactive. However, in epithelial contexts, FUT8 can also have protective roles. This is evident in its regulation of epithelial glycosylation programs and barrier maintenance pathways.

#### 1.2.3 Putative roles of α1,3-fucosyltransferase in IBD

Other fucosyltransferases, such as FUT3 and FUT7, are not as clearly described in the provided studies, but they likely play a role in shaping glycan diversity and immune cell movement through the synthesis of Lewis antigens and formation of selectin ligands. Their importance is indicated by the general finding that changes in fucosylation patterns in IBD affect how immune cells are located, how microbes settle, and how inflammatory signaling works. However, FUT2 and FUT8 are still the most clearly defined enzymes in experimental colitis.

### 1.3 Roles of fucosidase in IBD

Fucosidases are hydrolases that cleave off terminal fucose residues from glycoconjugates and have a regulatory role with regard to the availability of fucose residues, mucosa glycosylation, and interaction between the host and its microbiome. The research presented below is not directly aimed at manipulation of the genes of fucosidases in the host, although the data presented below may be useful to understand how glycans containing fucose residues are metabolized in the gut of an individual with IBD and how fucose-rich glycans affect the development and severity of colitis.

An interesting observation was made with regard to the effect of fucose-rich fucoidan preparations. Fucoidans are structural polymers rich in fucose residues; although they cannot be fully degraded due to their structure, they can be partially degraded by fucosidase activities of the enzymes of microbiome. Oral administration of fucoidan to mice with acute colitis (DSS-induced) ameliorated symptoms such as weight loss, diarrhea, and tissue damage, as well as reduced production of proinflammatory cytokines, improving the tissue histology (26). Moreover, there was down-regulation of the expression of inflammatory mediators in the colonic mucosa, implying that fucosidase-mediated metabolism of fucose-rich glycans contributed to anti-inflammatory action.

Recent studies also provide evidence for the idea that microbial metabolism of fucose-containing carbohydrates impacts gut homeostasis. In a colitis mouse model induced by DSS, the synbiotic approach of fucoidan derived from *Sargassum horneri* combined with *Lactobacillus plantarum* restored intestinal permeability and alleviated inflammation by modifying microbial communities and host signaling cascades (27). Fractions of fucoidan high in complex fucosylation stimulated the growth of bacteria and increased microbial diversity in the gut, while at the same time decreasing inflammation pathways (NF-κB, iNOS, COX2) and restoring tight junction proteins, such as ZO-1 and occludin.

Overall, fucose and fucosylation play critical roles in IBD pathogenesis by impacting the epithelial barrier, immune cell function, microbial colonization, and metabolism.

FUT2-driven α1,2-fucosylation mainly supports the strength of the epithelial barrier, the balance of microbes, and the repair of mucosal tissue. In contrast, FUT8-driven core fucosylation improves immune receptor signaling and inflammatory responses in both the innate and adaptive immune systems.

**Figure 1:**
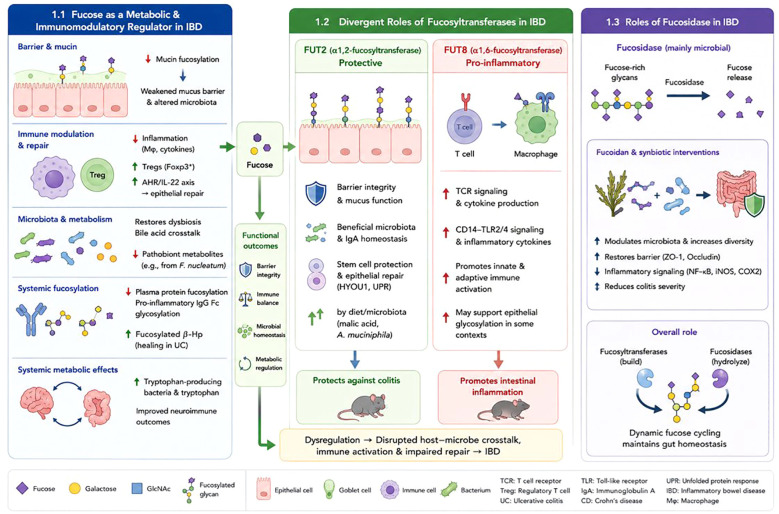
Roles of fucose metabolism and fucosylation pathways in inflammatory bowel disease (IBD). Fucose and fucosylated glycans regulate intestinal homeostasis through effects on epithelial barrier integrity, immune responses, and gut microbiota composition. Reduced mucosal and systemic fucosylation in IBD is associated with impaired mucus barrier function, dysbiosis, and pro-inflammatory immune activation. FUT2-mediated α1,2-fucosylation generally exerts protective effects by maintaining epithelial integrity, supporting IgA-mediated host–microbial interactions, and promoting intestinal stem cell function and repair. In contrast, FUT8-mediated core fucosylation enhances inflammatory signaling in T cells and macrophages through T-cell receptor (TCR) and TLR2/4-CD14 pathways, thereby promoting intestinal inflammation. Microbial fucosidases metabolize fucose-rich glycans such as fucoidan, generating beneficial effects on microbial diversity, epithelial tight junctions, and inflammatory signaling. Fucoidan-based synbiotic approaches further improve intestinal barrier function and reduce colitis severity. Overall, balanced fucose metabolism contributes to immune homeostasis and mucosal protection, whereas dysregulated fucosylation promotes IBD progression.

Fucose-metabolism-dependent modulation of fucosidase activity impacts the generation of fucose metabolites and microbiota ecology, thereby controlling downstream events including epithelial barrier function, cytokine release, and immune system regulation in colitis. Fucosyltransferases catalyze fucosylation reactions, while fucosidases are involved in the opposite pathway, thereby regulating fucose-metabolism-dependent processes. The interplay between both enzymatic activities may facilitate fucose metabolism in the intestinal tract, and the imbalance of both enzymes is likely associated with the pathophysiology of IBD, while pharmacological intervention involving fucose-rich molecules (such as fucoidans) leads to a normal microbiome and epithelial cell physiology, ameliorating colitis severity (**Figure 1**).

Box 1 **The Fucosyltransferase system as a multilayered glycosylation network**

Human fucosyltransferases are Golgi- or ER-localized glycosyltransferases that catalyze the transfer of fucose from GDP-fucose to glycoconjugates, generating structurally and functionally diverse fucosylated epitopes that regulate immunity, development, and disease. They are traditionally divided into α1,2- (FUT1/FUT2), α1,3/4- (FUT3-FUT7, FUT9), α1,6- (FUT8), and protein O-fucosyltransferases (POFUT1-4), each with distinct substrate specificity and biological roles.

α1,2-fucosyltransferases FUT1 and FUT2

The α1,2-fucosyltransferases FUT1 and FUT2 generate the H antigen, the structural foundation of ABO blood group antigens, with FUT1 acting mainly in erythroid/endothelial tissues and FUT2 in secretory epithelia and mucosa (28, 29). These enzymes shape blood group phenotypes (Bombay/para-Bombay), secretor status, and host-microbiome interactions (29). FUT2 additionally modulates mucosal glycans that influence microbial colonization and immune homeostasis (30).

α1,3-fucosyltransferase family (FUT3-FUT7, FUT9)

The α1,3-fucosyltransferase family (FUT3-FUT7, FUT9) produces Lewis antigens (Le^x^, Le^y^) and sialyl Lewis X, which regulate cell adhesion, immune trafficking, neuronal development, and cancer metastasis (31). FUT9 is the dominant enzyme for brain Le^x^ synthesis and neuronal glycosylation (32), while FUT7 is a key regulator of inflammatory leukocyte adhesion via sLe^x^ modification of endothelial ligands (33). Together, this family acts as a central regulator of immune-cell trafficking and developmental patterning.

α1,6-fucosyltransferase (FUT8)

The α1,6-fucosyltransferase FUT8 is the sole enzyme responsible for core fucosylation of N-glycans (34). This modification is critical for growth factor receptor signaling (EGFR, TGF-β), integrin function, and tumor progression (35). Structural studies reveal a highly specific catalytic mechanism involving an exosite required for branched N-glycan recognition (36), while functional studies confirm its necessity in cancer progression and its potential as a therapeutic target (35).

O-fucosyltransferases (POFUT1-POFUT4)

Finally, O-fucosyltransferases (POFUT1-POFUT4) catalyze protein domain-specific O-fucosylation rather than glycan remodeling. POFUT1 regulates Notch signaling and hematopoietic homeostasis via EGF-repeat modification (37), while POFUT2 modifies thrombospondin repeats involved in extracellular matrix protein maturation (38). Newly redefined enzymes POFUT3/4 (formerly FUT10/11) modify EMI domains in ER quality control pathways, expanding the scope of intracellular O-fucosylation (39). POFUT3 additionally supports stem cell maintenance and neural precursor migration through specialized Le^x^-related functions (40).

Together, these findings establish fucosyltransferases as a highly organized enzymatic network that integrates glycan and protein modification systems across cellular compartments. Rather than serving merely as structural glycan modifiers, FUT enzymes act as central regulators of cell signaling, immune recognition, developmental programming, and disease progression. Their diverse but coordinated activities underscore fucosylation as a key molecular language linking glycobiology to physiology and pathology.

## 2 Fucosylation in CRC

### 2.1 The role of fucose metabolism in colorectal cancer progression and diagnosis

Fucose and fucosylation are repeatedly highlighted across multiple layers of CRC biology, ranging from early detection biomarkers to functional regulators of tumor progression and therapeutic targeting. Collectively, the literature indicates that altered fucosylation is a consistent and multifaceted hallmark of CRC, involving both α1,2-, α1,3-, and α1,6-linked fucose structures, as well as changes in fucose metabolism. These alterations are observed not only in tumor tissues but also in circulating glycoproteins and extracellular vesicles, supporting their relevance for both tumor biology and clinical translation.

Early glycoproteomic profiling studies demonstrated that CRC and precancerous adenomas exhibit increased global fucosylation in plasma glycoproteins, often alongside elevated sialylation, suggesting that glycan remodeling occurs early during tumorigenesis (41). Specific circulating glycoproteins such as complement C3, histidine-rich glycoprotein, and kininogen-1 were identified as carriers of aberrant fucosylation, highlighting the potential of fucose-containing N-glycans as minimally invasive biomarkers for disease detection and stratification (41).

At the tissue level, the disruption of the fucose supply system shows its biological importance in CRC. The overexpression of the GDP-L-fucose transporter was significantly higher in tumor tissues compared to normal tissues. This suggests that greater fucose transport into the Golgi is a key step that leads to increased α1,6-fucosylation in CRC (42). Similarly, enzymes that control GDP-L-fucose production, such as TSTA3, are upregulated in CRC and directly contribute to higher cellular fucosylation. This increase promotes cancerous traits like growth, movement, invasion, and epithelial-mesenchymal transition (43). These findings show that higher fucosylation in CRC is not just a passive change; it is actively maintained through metabolic and enzymatic reprogramming.

Functionally, fucosylation contributes to tumor progression and interactions with the tumor microenvironment. Experimental studies show that changing fucose metabolism affects cancer cell behavior. Inhibiting fucosylation reduces the proliferation and metastatic potential of CRC cells (43). On the other hand, adding exogenous L-fucose can affect intracellular metabolism, including serine biosynthesis pathways. This process indirectly regulates fucosylation levels and impacts tumor growth, depending on the context (44). These findings suggest that fucosylation connects closely with larger metabolic networks. It links nutrient availability and glycan remodeling to the aggressiveness of tumors.

In parallel, fucosylation plays a key role in tumor-microbiome interactions. L-fucose has been shown to counteract the tumor-promoting effects of Fusobacterium nucleatum. It reduces STAT3 activation and epithelial-mesenchymal transition in CRC cells (45). This suggests that fucose-related pathways may influence inflammation-driven tumor growth and microbiota-related cancer signaling. From a clinical perspective, altered fucosylation is strongly linked to diagnostic and prognostic potential. Fucosylated forms of serum proteins, such as leucine-rich α-2-glycoprotein-1 (LRG1), which carry fucosylated triantennary N-glycans, show high diagnostic accuracy. They outperform traditional markers like CA19-9, especially when combined with CEA (46). These studies highlight that specific changes in fucosylation provide better clinical insights compared to conventional protein-based markers.

Fucosylation signatures are also present on tumor cell surfaces and extracellular vesicles. CRC cells and exosomes consistently show altered core fucosylation patterns (α1,6-fucose) and α1,3-linked fucosylated glycans that indicate their role in intercellular communication and tumor microenvironment remodeling (47). Exosomal fucosylation is also a promising target for liquid biopsy for early detection and monitoring of disease (48).

Further exploitation of these glycan changes for clinical applications has arisen from technological advances (49). Lectin-based systems targeting alpha-L-fucose for imaging premalignant lesions of colorectal neoplasia have been used for endoscopic detection of early colorectal neoplasia. Besides, lectin-functionalized magnetic nanoparticles and lectin-drug conjugates targeting fucosylated glycans are capable of efficient capture of circulating tumor cells and selective tumor killing, revealing the therapeutic and diagnostic potential of fucose targeting strategies (50, 51).

### 2.2 Divergent roles of fucosyltransferases in CRC

Fucosyltransferases (FUTs) are essential proteins involved in the incorporation of fucose into glycoconjugates, and numerous reports indicate that defective fucosylation plays a crucial role in the pathogenesis of CRC tumors, metastasis, immune function, and treatment outcomes. In different studies, various types of FUT genes (such as FUT2, FUT3, FUT4, FUT8, FUT9, and POFUT1/2) have been reported to mediate a range of oncogenic events by means of core (α1,6-) or terminal (α1,2/1,3/1,4-) fucosylation.

An important aspect of CRC development is mediated by the core fucosylation modification regulated by the FUT8 enzyme on N-linked glycans of signaling and immunologically relevant molecules. FUT8 dysregulation has been documented in CRCs and has a distinct impact depending on the cellular context. For instance, FUT8 regulates tumor cell growth, migration, and invasiveness through activation of Wnt/β-catenin signaling pathways as well as through regulation of LEF1-AS1/LEF1 transcription factors (52). FUT8 has been shown to affect apoptosis and chemotherapeutic drug resistance through ERK signaling and TRAIL mechanisms, impacting patient treatment response in a stage-specific fashion (53). In the setting of metastasis, FUT8 stabilizes B7-H3 checkpoint protein through core fucosylation and promotes immune escape, and pharmacologic inhibition of FUT8 leads to lysosomal degradation of B7-H3 and metastasis suppression (54). Proteomic and glycomic analysis revealed widespread remodeling of protein profiles and N-glycan modifications after depletion of FUT8 (55). Clinically, FUT8 expression has prognostic value in CRC, particularly in relation to p53 status (56), while inhibition of α1,6-fucosylation alters proliferation, migration, and adhesion in a tumor-stage-dependent manner (57).

Furthermore, in addition to FUT8, FUT2 serves a dual role in CRC progression and metastasis through α1,2-fucosylation and the regulation of Wnt signaling pathways. The elevated expression level of FUT2 in CRC cells enhances cancer formation and metastasis through the activation of the Wnt/β-catenin signaling pathway (58). Nonetheless, the expression of FUT2 can also serve as an inhibitor for the metastasis process by elevating the fucosylation level of LRP1 to inhibit EMT (59). From a mechanistic perspective, FUT2 not only participates in signal transduction but also mediates the metabolic reprogramming of CRC cells by facilitating the generation of fatty acids through the YAP/TAZ and SREBP1 signaling pathways (60).

FUT3 modulates EMT, stress adaptation, and signaling through TGF-β and ER stress pathways. FUT3 promotes fucosylation of TGFβ receptor I, enhancing TGF-β-induced EMT and metastatic potential (61). It also modifies GRP78 fucosylation, enabling activation of the PERK-eIF2α-ATF4 signaling pathway under glucose restriction, thereby supporting tumor survival in nutrient-deprived microenvironments (62). In addition, RNA-binding protein DDX39B enhances FUT3 expression, further linking RNA metabolism to glycosylation-driven EMT and metastasis (63). Together, these studies position FUT3 as a key mediator of EMT, stress resistance, and microenvironmental adaptation in CRC.

The role of FUT4 and FUT9 in extending the spectrum of biological functions of fucosylation in CRC can also be noted. Specifically, the activity of FUT4 is controlled via the exosomal MALAT1/miR-26a/26b axis, inducing α1,3-fucosylation and triggering PI3K/AKT/mTOR signaling in order to facilitate metastatic dissemination (64). In turn, FUT9 is highly associated with the characteristics of cancer stemness and chemoresistance, contributing to the development of stem cell-like properties in terms of CD44, ALDH, and Sox2 expression, as well as improved 5-FU resistance (65).

In addition to glycosylation of proteins that have been traditionally known for their function, fucosylation is involved in regulating immune responses and development via proteins belonging to the POFUT family. Mutations leading to enhanced O-fucosylation of NOTCH via gain of function of POFUT1 can cause abnormal NOTCH signaling in colorectal cancer (CRC) cells (66). On the other hand, fucosylation of JUP by POFUT2 results in higher expression of VEGFA and tumor angiogenesis (67).

In the clinical aspect, it is seen that multiple investigations have established the relationship of the fucosylation pattern of serum glycans and IgG with CRC diagnostics, stages, and prognosis. In this regard, the fucosylated forms of certain core proteins, such as fucosylated β-Hp and LRG-FTG have been used for their diagnosis/prognosis purposes (46, 68), whereas N-Glycan analysis of serum and IgG has revealed a number of variations in their core fucosylation, sialylation, and branches throughout CRC progression (69–71). Exosome-based analyses further show that core fucosylation by FUT8 changes are conserved in tumor-derived vesicles, supporting their potential use in liquid biopsy (72).

### 2.3 Fucosidase-mediated glycan editing in CRC

Fucosidases have been recently identified as modulators of glycan remodeling in CRC, contributing to biomarker generation and tumor cell behavior by modifying fucosylated glycans. Even though not as extensively investigated as their counterpart fucosyltransferases, it is now clear that fucosidase function and regulation have intimate connections with tumor-specific fucosylation, protein stability, and drug resistance.

Early mechanistic information related to the regulation of fucosidase-sensitive glycosylation of glycoproteins in CRC is the association of the α1,3/4 fucosylated β-Hp with colon cancer. In this study (73), using a combination of lectin binding and mass spectroscopy techniques, it was found that there is an increased degree of fucosylation at N241 site of β-Hp in cancer patients, compared to healthy subjects and those with inflammatory bowel disease. Interestingly, the fucosylated β-Hp was resistant only to the enzyme α1-3/4 fucosidase but not to α1,6 fucosidase, suggesting that fucosidase-sensitive glycoforms are highly specific in Fucα1-3/4GlcNAc modification. Moreover, the degree of expression of such a modification correlates with disease progression and declines post-treatment.

In recent studies, a role for fucosidase regulation in the pathophysiology of CRC has been established via the FUCA1 pathway. The FUCA1 gene codes for α-L-fucosidase 1, an enzyme involved in the removal of terminal fucose sugars from glycoconjugates. In CRC cells, FUCA1 was reported to be a substrate of the deubiquitinating enzyme USP35, which is overexpressed in CRC tumors (74). The stability of FUCA1 due to its interaction with USP35 is associated with promoting cell proliferation and resistance to chemotherapy drugs like oxaliplatin and 5-fluorouracil. At the mechanistic level, the FUCA1-USP35 interaction pathway upregulates the expression of NER pathway proteins (XPC, XPA, ERCC1), resulting in increased resistance to platinum-containing drugs.

Together, these studies clearly demonstrate that fucosyltransferases play integral roles in regulating complex signaling networks in colorectal cancer, including oncogenic pathways such as Wnt, TGF-β, PI3K/AKT, and ERK, as well as metabolic reprogramming, epithelial–mesenchymal transition (EMT), immune evasion, stemness, and angiogenesis. However, the precise effects of fucosylation on these signaling pathways appear to depend largely on the specific fucosyltransferase involved (e.g., FUT2, FUT3, or FUT8), the stage of tumor development, and the surrounding cellular microenvironment.

In contrast, fucosidases in colorectal cancer appear to function at two interconnected levels: (1) modulation of tumor-associated fucosylated glycoproteins at the biomarker level, where α1,3/4 fucosidase-sensitive structures such as β-Hp serve as diagnostic indicators (73), and (2) direct regulation of tumor biology through FUCA1-mediated remodeling of fucosylated substrates, thereby influencing cellular proliferation and chemotherapeutic responsiveness (74). Collectively, these findings suggest that fucosidase-mediated remodeling of fucosylation states represents an underappreciated yet biologically and clinically important layer of glycan regulation in colorectal cancer progression and treatment resistance.

## 3 Roles of fucose and fucosylated glycans in NEC

NEC is a critical inflammatory condition affecting the pre-term infant’s intestines that features epithelial damage, immune regulation dysfunction, microflora dysbiosis, and poor intestinal perfusion. Recent research points to the pivotal significance of fucose and fucosylated glycoproteins, especially those originating from the epithelium and human milk oligosaccharides (HMOs), in the processes of maintaining intestinal integrity and resisting NEC development. Overall, current findings demonstrate that both endogenous epithelial fucosylation and exogenous fucose-based substances modulate NEC-related pathways (10, 75–79).

The basic principle revolves around the process of fucosylation, which helps maintain the integrity of the intestinal epithelium. Fucosylation of glycans in IECs, mainly controlled by the FUT2 gene, is critical for the defense of the mucosa. There is a significant decrease in fucosylation levels in the epithelium in clinical samples and animal models of NEC, which correlates with reduced IL-22 levels and decreased ILC3s. Disruption of the IL-22-FUT2 pathway results in further damage to the intestines and increased inflammation, demonstrating that fucosylation defects in the epithelium increase NEC risk (75).

Another role that fucose plays is the promotion of epithelial repair and regeneration through ISCs. The consumption of L-fucose improves the proliferation of ISCs and epithelial cell renewal via indirect immune-dependent pathways. This is achieved by activating the AHR pathway in immune cells located in the lamina propria, resulting in the secretion of IL-22, which then enhances ISC stemness through IL-22R-STAT3 signaling and epithelial regeneration (10). Meanwhile, fucose exerts microbe-dependent functions through the alteration of the gut microbial composition by favoring the presence of *Akkermansia muciniphila* and promoting propionate synthesis. The microbes’ metabolites promote ISC function via GPR41/43 receptor activation and Wnt signaling pathways (76).

Furthermore, fucose plays an essential role in the maintenance of microbiome homeostasis by acting as a substrate that influences the gut microbiome and favors beneficial bacteria. Although alterations in microbiomes alone may not be sufficient for providing protection, the effects of microbial metabolism induced by fucose metabolism, especially SCFAs, along with signaling mechanisms of the host, can promote epithelial robustness and immunological balance (76).

## 4 Roles of 2′-fucosyllactose and 3-fucosyllactose in gut homeostasis, microbiota regulation, and intestinal inflammation

Another critical protective role of fucosylated glycans is the modulation of innate immune signaling. HMOs, particularly the fucosylated glycans 2′-FL and 3-fucosyllactose (3-FL), are increasingly recognized as multifunctional bioactive molecules that shape gut microbial ecology, strengthen epithelial barrier integrity, and modulate inflammatory signaling. These oligosaccharides are abundant in human milk and act as key regulators of early-life microbial colonization and intestinal immune development, while emerging evidence demonstrates their therapeutic relevance in IBD across developmental stages (80).

### 4.1 Metabolic programming and microbiota modulation

One of the key functions of 2′-FL is its prebiotic activity, selectively promoting beneficial taxa such as *Bifidobacterium*, *Lactobacillus*, and butyrate-producing commensals. In adult and disease-associated microbiota, 2′-FL consistently enhanced SCFA production, especially acetate and propionate, and enhanced microbial metabolic output compared with conventional prebiotics (81). In UC-associated microbiota, 2’-FL also downregulates pro-inflammatory taxa such as *Desulfovibrio* spp. while promoting SCFA production, suggesting disease-specific microbiome remodeling (81).

Mechanistically, 2’-FL supports trophic microbial networks where bifidobacteria degrade fucosylated substrates into metabolites such as lactate, acetate, and 1,2-propanediol that are further converted by secondary fermenters (e.g. *Eubacterium hallii*) into butyrate and propionate (82). These cross-feeding interactions show how 2′-FL promotes ecosystem-level metabolic cooperation that improves intestinal homeostasis.

*In vivo* and *ex vivo* studies further confirm that 2′-FL shapes gut microbial communities toward increased abundance of *Bifidobacterium*, *Lactobacillus*, and *Faecalibacterium prausnitzii*, while restoring dysbiotic profiles in IBD and pediatric CD models (83–85).

### 4.2 Barrier protection and epithelial integrity

Besides microbiota modulation, 2′-FL and 3-FL directly boost intestinal epithelial barrier integrity. Both fucosyllactoses restore the expression of tight junction proteins, such as ZO-1 and occludin, in IL-6 and DSS-induced colitis models, decrease epithelial permeability, and maintain colon architecture (86). In models of epithelial injury caused by NSAID, 2′-FL also preserves F-actin organization and tight junction stability in neuro-epithelial cocultures, indicating microbiota-independent barrier protection (87). Furthermore, 2′-FL improves mucosal barrier function by helping the recovery of goblet cells and raising MUC2 expression, thus strengthening the mucus layer (88). This mucus-enhancing activity is accompanied by the regulation of NLRP6, a major modulator of epithelial immunity and TLR4/NF-κB signaling.

2′-FL and 3-FL directly enhance intestinal epithelial barrier integrity independent of microbiota modulation. Both fucosyllactoses are able to restore tight junction proteins such as ZO-1 and occludin, reduce epithelial permeability, and sustain colon architecture in IL-6 and DSS-induced colitis models (86). 2′-FL also preserves F-actin organization and tight junction stability in neuro-epithelial cocultures in NSAID-induced epithelial injury models, demonstrating microbiota-independent barrier protection (87).

Moreover, 2′-FL improves mucosal barrier function by promoting the recovery of goblet cells and increasing the expression of MUC2, thereby enhancing the mucus layer (88). The mucus-promoting activity correlates with modulation of NLRP6, an important regulator of epithelial immunity and TLR4/NF-κB signaling.

### 4.3 Pathways of anti-inflammatory signaling

Emerging evidence suggests that 2′-FL and 3-FL exert direct anti-inflammatory effects via multiple signaling pathways. Both oligosaccharides suppress IL-6-, TNFα- and NF-κB-driven inflammation in epithelial and immune cells (86). 2′-FL and 6′-sialyllactose inhibit epithelial apoptosis and inflammatory injury in NEC models by direct engagement with the TLR4-MD2 complex and TLR4 signaling (77).

2′-FL suppresses STAT3 phosphorylation and palmitoylation, reduces mucosal inflammation, and restores barrier function in UC (89). Other studies report that 2′-FL modulates NLRP6-TLR4 axis signaling and inhibits NF-κB activation, integrating the epithelial and immune regulatory pathways (88).

### 4.4 Protection in NEC and ischemic injury

In mouse and porcine models of neonatal disease, 2′-FL consistently ameliorates the severity of NEC. Mechanistically, it enhances intestinal perfusion through eNOS-dependent pathways, improving mesenteric blood flow and preventing ischemic injury (78). In parallel, 2′-FL reduces TLR4-driven inflammation and epithelial apoptosis in NEC models in both mice and piglets (77). However, its protective efficacy may depend on developmental timing and microbiota maturity, as early postnatal short-term supplementation alone may be insufficient to alter disease outcomes (79).

### 4.5 Microbial metabolism and implications for adult disease

Evidence that has recently emerged expands the role of 2′-FL into adulthood and demonstrates that it modulates microbial metabolites and stimulates the production of bioactive metabolites, including pantothenol and pantothenate, which improve epithelial resilience under oxidative stress (90). These microbiota-derived metabolites are associated with protection against colitis and restoration of intestinal homeostasis.

## 5 Fucose-microbiota-SCFA-tight junction axis in intestinal homeostasis

The gut microbiota influences intestinal barrier function and the host immune system, thereby regulating glucose and lipid metabolism (91). Fucosylation in host and fucose metabolism by gut form a central regulatory axis linking epithelial integrity, immune responses, microbial ecology, and disease progression in the gut. Across IBD, CRC, and NEC, balanced fucosylation supports barrier function, immune tolerance, and microbial homeostasis, whereas its dysregulation drives inflammation, dysbiosis, and tumor progression. Fucosyltransferases and fucosidases coordinate dynamic glycan remodeling that influences signaling pathways, metabolic adaptation, and host-microbiota interactions.

Fucose is a host-derived glycan, and a nutrient source for microorganisms, which promotes the growth and metabolic activity of beneficial gut bacteria. Host intestinal tract fucosylation is dynamically regulated in host-microbe interactions as an ecological signal for symbiosis promotion and pathogen virulence suppression (92). Experimental studies have demonstrated that fucose administration changes the microbial community by increasing the abundance of *Akkermansia muciniphila* and *Bifidobacterium*, which are strongly linked to SCFA metabolism (14, 76). Fucose metabolism is interesting because intermediate metabolites such as 1,2-propanediol and lactate are produced. These serve as substrates for cross feeding networks of microbes resulting in increased SCFAs production, particularly propionate (93). These metabolic interactions highlight that fucose is not acting in isolation but rather as an upstream regulator of microbial fermentation pathways converging on SCFA synthesis.

SCFAs (acetate, propionate and butyrate) are well known microbial metabolites that regulate epithelium and immune homeostasis. Their mechanisms of action include activation of GPR41 and GPR43, inhibition of inflammatory signaling pathways and regulation of energy metabolism in epithelial cells. These SCFAs have repeatedly been shown to promote intestinal barrier function and inhibit inflammation (94, 95). In addition to the SCFA-mediated effects, fucose can directly maintain and strengthen the integrity of the epithelial barrier through mechanisms including host glycan modification and microbiota alteration. In epithelial cells fucose is involved in FUT2-dependent fucosylation that strengthens the mucosal surface and promotes the enhanced expression and stabilization of tight junction constituent proteins such as ZO-1, occludin and claudins (92). In addition, the fucose-induced remodeling of the microbiota prevents the colonization by pro-inflammatory or barrier-disrupting pathogens (e.g., *Fusobacterium* spp.) and inhibits the production of damaging microbial metabolites that damage tight junctions (14, 92). These effects are further enhanced by improved integrity of the mucus layer and function of goblet cells, thus protecting the junctional complexes between epithelial cells.

Furthermore, fucose modulates tryptophan metabolism not by direct biochemical transformation but by remodeling the microbiota composition. During inflammation, fucose supplementation restores the population of tryptophan-producing bacteria (e.g. *E. coli*) and normalizes systemic tryptophan levels (14). Additionally, fucose increases microbial production of tryptophan-derived indole metabolites, with *Akkermansia muciniphila* contributing to the production of compounds such as indole-3-ethanol (96). These indole compounds activate the aryl hydrocarbon receptor (AhR) promoting IL-22 mediated mucosal healing and immune homeostasis.

## 6 Mechanisms of aberrant fucosylation-related intestinal inflammation

Some gut bacteria cleave terminal fucoses that are linked to glycoprotein (e.g. mucin) with α1,2- or α1,3-linkage. Bacteria and others can take up free fucoses.

Aberrant fucosylation contributes to intestinal inflammation, primarily through the disruption of host-microbiota interactions and the modulation of immune responses. FUT2-dependent α1,2-fucosylation dictates the composition and metabolic activity of the gut microbiota; loss-of-function mutations in FUT2 (non-secretor status) are associated with alterations in microbial communities, reduced bacterial loads on mucosal surfaces, subclinical inflammation, and an increased risk of CD (97). Mechanistically, the reduction of fucosylated glycoconjugates in FUT2-deficient individuals inhibits the colonization of commensal bacteria, such as those of the genus *Escherichia*, thereby allowing the expansion of pro-inflammatory bacteria that trigger inflammatory T-cell responses (98). Similarly, mice lacking fucosylation exhibit heightened susceptibility to enteric pathogens such as *Citrobacter rodentium*, *Salmonella typhimurium*, and the resident bacterium *Escherichia coli*, resulting in exacerbated intestinal inflammation (99–101). Furthermore, microbiota alterations in FUT2-knockout mice promote the production of lysophosphatidylcholine, which compromises tight junction integrity and enhances the production of inflammatory cytokines (19). In contrast to FUT2 deficiency, increased FUT8 expression in inflamed colonic tissue from patients with ulcerative colitis is associated with increased mucin secretion and increased bacterial adhesion to and invasion of the epithelium, suggesting that both a deficiency and an excess of fucosylation may contribute to the pathogenesis of the disease (102).

Fucosylation also influences intestinal immunity by regulating microbe-specific T-cell populations and innate immune signaling pathways. In non-secretor individuals, there is an increased frequency of CD8^+^ T cells associated with the genera *Alistipes* and *Phascolarctobacterium*, as well as Th17 cells associated with the *Erysipelotrichaceae* family UCG-003; these changes may promote the development of inflammatory bowel disease (98). In contrast, wild-type FUT2 is associated with anti-inflammatory DP8α regulatory T cells induced by *Faecalibacterium prausnitzii* (103). Among fucosyltransferases, FUT8-mediated core fucosylation plays a central role in immune regulation. While core fucosylation is increased in T cells derived from inflamed mucosa, FUT8 deficiency inhibits T-cell receptor signaling and ameliorates experimental colitis (24). Similarly, the loss of core fucosylation in macrophages suppresses CD14-dependent TLR4 and TLR2 signaling, reduces the production of inflammatory cytokines, and exerts a protective effect against DSS-induced colitis (25). Furthermore, while FUT7 promotes leukocyte recruitment via selectin-ligand interactions, FUT7 deficiency alleviates experimental colitis by limiting the migration of inflammatory cells (104).

## 7 Future direction: potential fucosylation-related therapy approaches in inflammatory diseases in gut

The impaired fucosylation of the intestinal epithelium is associated with barrier damage and microbiome dysbiosis, and thus increasing the exogenous fucosylation pathway might be helpful. In the mouse model of colitis, an oral supplement of l-fucose to activate the salvage synthetic pathway of fucosylation protects the intestinal epithelial cells from inflammatory injury. Duan et al. found that l-fucose administration in the dextran sulphate sodium-induced colitis mouse model significantly reduced the severity of inflammation and mortality of mice. Exogenous l-fucose increased the expression of metabolic enzymes in salvage pathway, such as fucose kinase and GDP-fucose-pyrophosphorylase in intestinal epithelial cells and enhanced the global fucosylation in intestinal epithelial cells and intestinal stem cells in mice. Moreover, FUT2 knockout impaired the protective effects of l-fucose and inhibited the fucosylation, which disrupted the unfolded protein response and induced excessive endoplasmic reticulum stress in intestinal stem cells. These results indicate the significant role of FUT2 in the fucose metabolism of the intestinal epithelium (20, 105). Also, l-fucose can alleviate the excessive accumulation of intestinal bile acid (such as Tauro-β-muricholic acid) and restore the compromised regulation of hepatic bile acid synthesis by regulating the bacteria structure, especially by increasing the abundance of *Lactobacillus*, thereby ameliorating the impairment of intestinal epithelium in dextran sulphate sodium-induced colitis (11).

Notably, fucose-derived glycans such as 2′-FL and 3-FL exert protective effects by restoring microbial balance, enhancing epithelial repair, and suppressing inflammatory signaling. Clinical and translational studies indicate that 2′-FL ameliorates gastrointestinal symptoms and microbial composition in IBS and UC patients, increasing SCFA production and beneficial taxa abundance (83). Comparative studies reveal that 2′-FL often outperforms conventional prebiotics (FOS, GOS) in SCFA induction, pathogen suppression by microbes, and immune modulation (81). Pediatric IBD models also demonstrate potent bifidogenic and anti-inflammatory effects of 2′-FL-containing formulations (84). Abbott which is a health care company reported that compared with infants receiving the control formula, both breastfed infants and those fed 2’-FL had significantly lower circulating concentrations of several pro-inflammatory cytokines, including IL-1 receptor antagonist (IL-1ra), IL-1α, IL-1β, IL-6, and TNFα, with reductions ranging from 29% to 83% (106). It suggests that infant formula including 2’-FL can protect from NEC. In parallel, aberrant fucosylation in CRC provides both mechanistic insight into tumor biology and clinically useful biomarkers.

Overall, fucose-centered glycosylation represents a targetable pathway in intestinal disease, offering promising opportunities for diagnosis, prognosis, and therapeutic intervention.

## Glossary

2′-FL: 2’-fucosylllactose
3-FL: 3-fucosylllactose
5-FU: 5-fluorouracil
AHR: aryl hydrocarbon receptor
AKT: protein kinase B (PKB)
ALDH: aldehyde dehydrogenase
ATF4: activating transcription factor 4
B7-H3: B7 homolog 3 protein (CD276)
CA19-9: cancer antigen 19-9
CD: Crohn’s disease
CD14: cluster of differentiation 14
CD44: cluster of differentiation 44
CEA: carcinoembryonic antigen
complement C3: complement component 3
COX2: cyclooxygenase 2
CRC: colorectal cancer
DDX39B: DExD-box helicase 39B
DNA: deoxyribonucleic acid
DSS: dextran sulfate sodium
EGF: epidermal growth factor
EGFR: epidermal growth factor receptor
eIF2α: eukaryotic translation initiation factor 2 alpha subunit
EMT: epithelial-to-mesenchymal transition
eNOS: endothelial nitric oxide synthetase
ER: endoplasmic reticulum
ERCC1: excision repair cross complementary gene 1
ERK: extracellular signal-regulated kinase
Foxp3⁺: forkhead box protein P3 positive
FUCA1: tissue alpha-L-fucosidase/alpha-L-fucosidase 1
FUT: fucosyltransferase
GPR41: G protein-coupled receptor 41
GPR43: G protein-coupled receptor 43
GDP: guanosine diphosphate
HMO: human milk oligosaccharides
HYOU1: hypoxia up-regulated 1
IBD: inflammatory bowel disease
IEC: intestinal epithelial cell
IgA: immunoglobulin A
IgG: immunoglobulin G
IL-1α: interleukin-1 alpha
IL-1β: interleukin-1 beta
IL-1ra: IL-1 receptor antagonist
IL-22: interleukin-22
IL-22R: interleukin-22 receptor
IL-6: interleukin-6
iNOS: inducible nitric oxide synthetase
ISC: intestinal stem cell
JUP: junction plakoglobin
LEF1: lymphoid enhancer-binding factor 1
LEF1-AS1: LEF1 antisense ribonucleic acid 1
Leˣ: Lewis X antigen
Leʸ: Lewis Y antigen
LRG1: leucine-rich α-2-glycoprotein-1
LRG-FTG: leucine-rich alpha-2-glycoprotein-1 with fucosylated triantennary N-glycan
MALAT1: metastasis associated lung adenocarcinoma transcript 1
miR-26a: micro ribonucleic acid-26a
miR-26b: micro ribonucleic acid-26b
mTOR: mammalian target of rapamycin
MUC2: mucin 2
NEC: necrotizing enterocolitis
NER: nucleotide excision repair
NF-κB: nuclear factor kappa-light-chain-enhancer of activated B cells
NHE3: Na(+)/H(+)-exchanger isoform 3
NLRP6: NLR family pyrin domain containing 6
NSAID: nonsteroidal anti-inflammatory drugs
PERK: protein kinase R-like endoplasmic reticulum kinase
PI3K: phosphatidylinositol 3-kinase
POFUT: O-fucosyltransferases
RNA: ribonucleic acid
SCFA: short-chain fatty acid
sLeˣ: sialyl-Lewis X antigen
Sox2: SRY-box transcription factor 2
SREBP1: sterol regulatory element-binding transcription factor 1
STAT3: signal transducer and activator of transcription 3
TAZ: Tafazzin
TGF-β: transforming growth factor-beta
TLR2: toll-like receptor 2
TLR4: toll-like receptor 4
TNFα: tumor necrosis factor alpha
TRAIL: TNF-related apoptosis-inducing ligand
Treg: regulatory T cell
TSTA3: tissue specific transplantation antigen P35B [GDP-L-fucose synthase (GFUS)]
UC: ulcerative colitis
USP35: Ubiquitin specific peptidase 35
VEGFA: vascular endothelial growth factor A
Wnt: wingless-related integration site
XPA: xeroderma pigmentosum, complementation group A
XPC: xeroderma pigmentosum, complementation group C
YAP: Yes-associated protein
ZO-1: zonula occludens-1
β-Hp: β-haptoglobin
